# A Novel Glucagon-Related Peptide (GCRP) and Its Receptor GCRPR Account for Coevolution of Their Family Members in Vertebrates

**DOI:** 10.1371/journal.pone.0065420

**Published:** 2013-06-11

**Authors:** Cho Rong Park, Mi Jin Moon, Sumi Park, Dong-Kyu Kim, Eun Bee Cho, Robert Peter Millar, Jong-Ik Hwang, Jae Young Seong

**Affiliations:** 1 Laboratory of G-protein Coupled Receptors, Graduate School of Medicine Korea University, Seoul, Republic of Korea; 2 Mammal Research Institute, Department of Zoology & Entomology, University of Pretoria, Hatfield, South Africa; 3 Medical Research Council Receptor Biology Unit, University of Cape Town, Observatory 7925, South Africa; 4 Centre for Integrative Physiology, University of Edinburgh, Edinburgh, Scotland; Van Andel Research Institute, United States of America

## Abstract

The glucagon (GCG) peptide family consists of GCG, glucagon-like peptide 1 (GLP1), and GLP2, which are derived from a common GCG precursor, and the glucose-dependent insulinotropic polypeptide (GIP). These peptides interact with cognate receptors, GCGR, GLP1R, GLP2R, and GIPR, which belong to the secretin-like G protein-coupled receptor (GPCR) family. We used bioinformatics to identify genes encoding a novel GCG-related peptide (GCRP) and its cognate receptor, GCRPR. The *GCRP* and *GCRPR* genes were found in representative tetrapod taxa such as anole lizard, chicken, and *Xenopus*, and in teleosts including medaka, fugu, tetraodon, and stickleback. However, they were not present in mammals and zebrafish. Phylogenetic and genome synteny analyses showed that *GCRP* emerged through two rounds of whole genome duplication (2R) during early vertebrate evolution. *GCRPR* appears to have arisen by local tandem gene duplications from a common ancestor of *GCRPR*, *GCGR*, and *GLP2R* after 2R. Biochemical ligand-receptor interaction analyses revealed that GCRP had the highest affinity for GCRPR in comparison to other GCGR family members. Stimulation of chicken, *Xenopus*, and medaka GCRPRs activated Gα_s_-mediated signaling. In contrast to chicken and *Xenopus* GCRPRs, medaka GCRPR also induced Gα_q/11_-mediated signaling. Chimeric peptides and receptors showed that the K^16^M^17^K^18^ and G^16^Q^17^A^18^ motifs in GCRP and GLP1, respectively, may at least in part contribute to specific recognition of their cognate receptors through interaction with the receptor core domain. In conclusion, we present novel data demonstrating that *GCRP* and *GCRPR* evolved through gene/genome duplications followed by specific modifications that conferred selective recognition to this ligand-receptor pair.

## Introduction

Glucagon (GCG) and GCG-like peptides exhibit a variety of functions in the brain, gut, and endocrine tissues [1]. The *GCG* gene encodes a large GCG precursor, which undergoes tissue-specific posttranslational proteolytic processing to produce mature GCG, glucagon-like peptide 1 (GLP1), and glucagon-like peptide 2 (GLP2) [2], [3], [4]. The mature form of GCG is released from the pancreatic islets of Langerhans α cells in response to low blood glucose level. Mature GCG enhances hepatic secretion of glucose by increasing glycogenolysis and gluconeogenesis in the liver [5]. GLP1 and GLP2 are produced in the intestinal L-type endocrine cells in response to food ingestion. GLP1 stimulates insulin secretion from pancreatic β cells in a glucose-dependent manner [6], [7]. GLP2 is a nutrient-responsive growth factor that stimulates specific trophic effects in the small and large intestines [8]. In the brain GLP1 and GLP2 are produced predominantly in brainstem neurons and transported to diverse regions of the central nervous system, including the hypothalamus, thalamus, and cortex [9], [10], [11]. GLP1 and GLP2 may increase satiety, leading to reduced nutrient consumption and weight loss [12], [13], [14]. In addition, GLP1 is neuroprotective and involved in neurite growth and spatial learning ability [15], [16]. The other GCC-related peptide is glucose-dependent insulinotropic peptide (GIP), which is encoded by the *GIP* gene. GIP is secreted from the K-cells of the upper intestine, duodenum and jejunum [17]. GLP1 and GIP are the main mammalian incretin hormones, accounting for approximately 50–70% of the total insulin secretion from pancreatic β cells [18]. In addition to its incretin effect, GIP has been implicated in lipid metabolism and the development of obesity via direct effects on adipose tissue. Further, GIP has been shown to promote bone formation by stimulating osteoblast proliferation and inhibiting apoptosis [19], [20].

These peptides exert their actions through the class B (or secretin-like) family of G protein-coupled receptors (GPCR) [21], [22]. Structural features of this family include a relatively long N-terminal extracellular domain (ECD), which contains an α-helix followed by four β-strands that form two antiparallel sheets [23], [24]. Six conserved cysteine residues lock these secondary structural elements together. In addition, an internal salt bridge called the Sushi domain further stabilizes this core structure [25]. Because each class B GPCR contains this conserved fold in the ECD, a common mechanism may underlie ligand recognition [26], [27], [28], [29]. Likewise, GCG family peptides share common structural elements, such as a random coiled N-terminus followed by an α-helix in the middle of each peptide [24], [30]. Crystal structures show that amino acids in the second half of the α-helix of GCG peptides interact with the N-terminal ECD. In particular, the hydrophobic face (Phe^22^, Ile/Val^23^, and Leu^26^) of the α-helix is highly conserved amongst GCG peptide family members [28]. These hydrophobic residues are exposed to the complementary hydrophobic binding pocket in the ECD [23], [24]. In contrast, the N-terminus and the first half of the α-helix of GCG peptides are believed to interact with the core domain of their respective receptors. The receptor core domain consists of 7 transmembrane helices and extracellular loops [27]. However, the crystal structure of the ligand-bound receptor core domain has not been reported. Therefore, despite extensive biochemical studies [31], [32], [33], [34], [35], [36], [37], [38], the ligand-binding residues within the core domain remain poorly defined. Recently, we reported that the evolutionarily conserved amino acid residues His^1^ and Thr^7^ in GLP1 and Ile^196^, Leu^232^, and Met^233^ in the core domain of GLP1R mediate selective ligand-receptor interaction and receptor activation [27], [39].

Increased access to genomic sequencing data for many vertebrate and invertebrate species and advances in bioinformatics have allowed identification of genes encoding novel peptides and GPCRs with BLAST search tools [40], [41], [42], [43]. In addition, synteny analyses and reconstruction of ancestral genomes have enabled exploration into the origin and relationship of peptide and receptor families [40], [44]. These analyses revealed that most peptide and receptor families expanded through two rounds of whole genome duplication (2R) and local duplications before and after 2R [40], [41], [42], [45]. These duplication processes are followed by modification and/or loss of genes. Ortholog-specific changes in the amino acid sequences of peptides and receptors may discriminate against interactions of a peptide with paralogs of the authentic receptor and promote selective interactions between a peptide family and its corresponding receptor family [27], [28].

In the current study, we performed a BLAST search in combination with genome comparison analysis and identified a gene encoding a novel glucagon-related peptide (GCRP) and its corresponding receptor (GCRPR) in various vertebrates. A previous study referred to GCRP and GCRPR as exendin and glucagon receptor-like receptor (GRLR), respectively [46]. However, our current phylogenetic and pharmacological studies revealed that GCRP is independent from the exendin lineage and has the highest affinity for GCRPR. Thus, we propose changing the names of the peptide and receptor to GCRP and GCRPR, respectively. We present an evolutionary history of GCRP and GCRPR based on phylogenetic and synteny analyses. In addition, we demonstrate the pharmacological properties of this ligand-receptor pair by performing ligand-binding, receptor activation, and signaling assays in a heterologous expression system. Finally, we identified amino acid residues within peptides that mediated selective interaction with their chimeric receptors.

## Results

### Presence of a Novel GCRP

**Figure 1:**
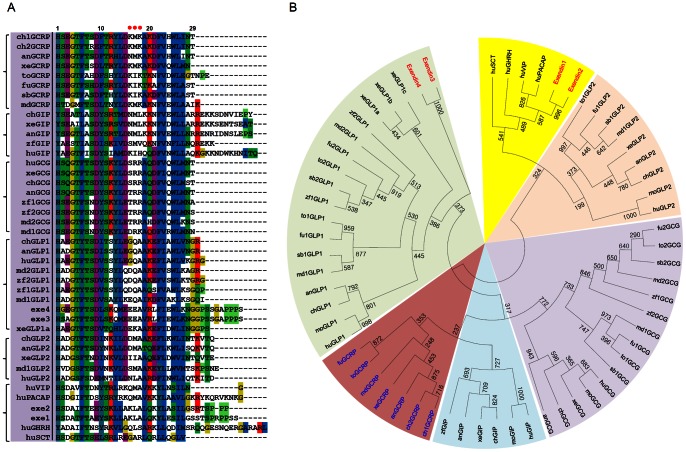
Amino acid sequence alignment of GCRP and neighbor-joining phylogenetic tree for related peptides of vertebrates. ***A,*** The mature peptide sequences of GCRP were predicted and aligned along with the GIP, GCG, GLP1, GLP2, and Gila monster exendins. Conserved residues for GCRPs and related peptides are indicated by different colors as proposed by the ClustalX-2.1 program. The GCRP-specific motif at positions 16–18 is highlighted by red dots above the sequences. ***B,*** Neighbor-joining phylogenetic tree for GCRP-related peptides of human (hu), mouse (mo), chicken (ch), anole lizard (an), *Xenopus (xe)*, zebrafish (zf), medaka (md), fugu (fu), stickleback (sb), and tetraodon (to) along with Gila monster exendins and human SCT, GHRH, VIP, and PACAP. The mature peptide sequences were aligned on ClustalX-2.1, and a tree was constructed with MEGA 5.05. Bootstrap numbers represent 1,000 replicates.

A genome BLAST search identified genomic fragments containing novel GCRP sequences in a variety of vertebrates, including chicken, anole lizard, *Xenopus tropicalis*, medaka, fugu, stickleback, and tetraodon but not in human, mouse, and zebrafish. Full-length cDNA sequences were available for the GCRP precursors in chicken and *Xenopus* [46]. However, only genome fragments having GCRP were identified for other species in our study. The predicted GCRP mature peptide sequence was distinct, while it retained a high degree of identity with GCG, GLP1, GLP2, and GIP. For example, the conserved KM/IK motif at positions 16, 17 and 18 was unique to GCRP, whereas other residues in GCRP are very similar to those found in this peptide family (Fig. 1A). A previous study by Irwin and Prentice [46] suggested that GCRP has a close relationship with exendin-4, which was originally discovered in the Gila monster (*Heloderma suspectum*) [47]. However, our phylogenetic study suggested that the amino acid sequences of exendin-4 and -3 were more similar to GLP1 than GCRP. In addition, exendin-1 and -2 have close relationships with VIP and PACAP. Thus, it seems likely that this novel GCRP belongs to a lineage independent of exendin or its related peptides.

Genome synteny analyses for *GCRP*-containing genomic fragments were described in our previous article [40]. *GCRP* was located near phosphodiesterase 1B (*PDE1B*), homeobox C13 (*HOXC13*), v-erb-b2 erythroblastic leukemia viral oncogene homolog 3 (*ERBB3*), insulin-like growth factor binding protein 6 (*IGFBP6*), myosin light chain 6B (*MYL6B*), and complement component 1 q subcomponent-like 4 (*C1QL6*) genes (Fig. S1A). However, the *GCRP* gene was not located on the human and zebrafish chromosomes that harbor these neighboring genes. There appear to be two forms of *GCRP* in chickens. One is located at 62 megabases of Un_random (UR), and the other is located at 1 megabase of the E22C19W28 fragment. These two genes may have emerged by a local tandem duplication, because *GCRP* neighbors in chicken E22C19W28 including aquaporin 2 (*AQP2*), Fas apoptotic inhibitory molecule 2 (*FAIM2*), and solute carrier family 4 sodium bicarbonate cotransporter member 8 (*SLC4A8*) were localized to *GCRP*-containing chromosomes in other vertebrates (Fig. S1A). Teleost-specific genome duplication may not have contributed to evolution of the second form of *GCRP*. *GCRP* may have emerged through two rounds of whole genome duplication, as the paralogs for *GCRP* and its neighbors were found to be aligned in three different paralogous chromosomal regions (Fig. S1B). Further details are provided in Information S1.

### Presence of a Novel GCRPR

**Figure 2:**
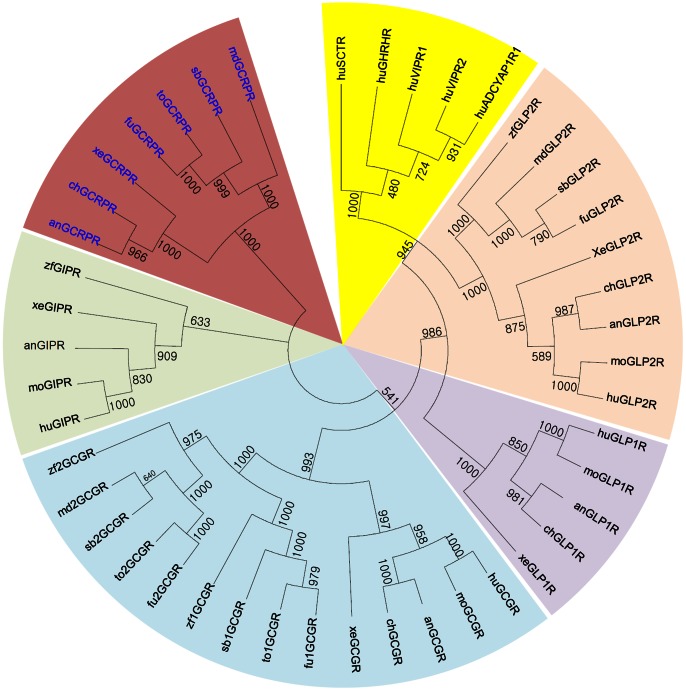
Neighbor-joining phylogenetic tree for GCRPR-related receptors. Human (hu), mouse (mo), chicken (ch), anole lizard (an), *Xenopus (xe)*, zebrafish (zf), medaka (md), fugu (fu), stickleback (sb), and tetraodon (to) along with human SCTR, GHRHR, VIPRs, and ADCYAP1R1 were examined. The amino acid sequences were aligned on ClustalX-2.1, and a tree was constructed with MEGA 5.05. Bootstrap numbers represent 1,000 replicates.

A genome BLAST search identified novel receptor sequences similar to those of GIPR, GCGR, GLP1R, and GLP2R in the genomes of chicken, anole lizard, *Xenopus tropicalis*, medaka, stickleback, tetraodon, and fugu. We named these sequences GCRPR. Thus, species that express GCRP also express the corresponding receptor. Phylogenetic analysis of GCRPR with its related receptors revealed that GCRPR has the closest relationship with GIPR (Fig. 2). The medaka and fugu GIPRs reported in a previous study [48] were redefined to be GCRPR in this study. The confusion in nomenclature for medaka and fugu GCRPRs is likely due to a phylogenetically close relationship between GCRPR and GIPR. Our phylogenetic and synteny analyses did not find GIP orthologs for medaka and fugu, whereas this species contained GCRP orthologs (Fig. 1 and Fig. S1). Interestingly, zebrafish contained both GIP and GIPR but lacked GCRP and GCRPR. These results indicate species-specific coevolution of the GCRP-GCRPR pair.

We previously described genome synteny analyses for GCRPR-containing genomic fragments [40]. Synteny analyses for genomic fragments containing *GCRPR* and its related receptors are very complicated. Thus, a detailed description is provided in Information S1. Briefly, our study showed that *GCRPR* clustered with *GCGR* and *GLP2R* on the same chromosome in many vertebrates, suggesting that these genes have arisen through local duplications after 2R. In contrast, *GLP1R* and *GIPR* were located on different chromosomes (Fig. S2). Neighbor gene analyses revealed that ohnologs (or paralogs) of *GCRPR/GLP2R/GCGR* neighbors, such as glutamate receptor ionotropic N-methyl D-aspartate 2C (*GRIN2C*), protein phosphatase 1 regulatory subunit 27 (*PPP1R27*), sirtuin 7 (*SIRT7*), peripheral myelin protein 22 (*PMP22*), lectin galactoside-binding soluble 9C (*LGALS9C*), and *FAM83E* were found on *GIPR*-containing genome fragments, suggesting that *GCRPR/GLP2R/GCGR*- and *GIPR*-containing genome fragments were generated by 2R. In addition, ohnologs (or paralogs) of *GIPR* neighbor genes DEAH box polypeptide 34 (*DHX34*), potassium channel subfamily K member 6 (*KCNK6*), and nuclear factor of kappa light polypeptide gene enhancer in B-cells inhibitor beta (*NFKBIB*) were observed in *GLP1R*-containing genome fragments (Fig. S2). This observation raises the possibility that one chromosome fragment harboring *GCRPR/GLP2R/GCGR* and *GLP1R* split into two chromosomal fragments before 2R. Thus, *GCRPR* appears to have emerged through local duplication of an ancestral gene of *GCRPR*, *GLP2R*, *GCGR*, and possibly *GLP1R*. However, further investigation is needed.

### GCRPs Activate GCRPRs with a Higher Potency than Related Peptides

**Figure 3:**
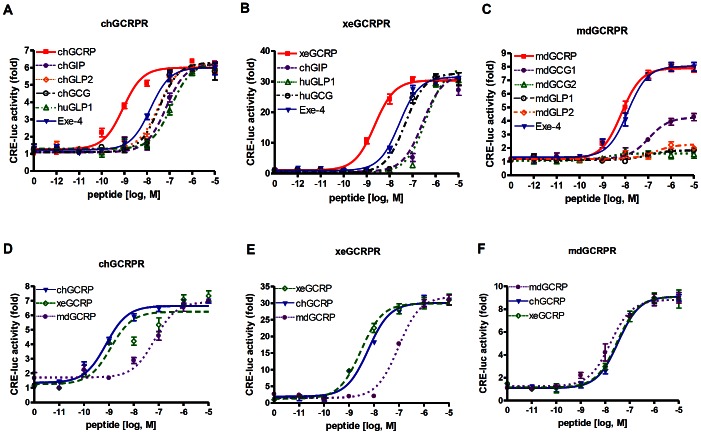
Activities of GCG family peptides on GCRP receptor. HEK293T cells were co-transfected with CRE-luc and plasmids containing chGCRPR (A), xeGCRPR (B), or mdGCRPR (C) in 48-well plates. Forty-eight hours after transfection, cells were treated with the indicated concentrations of peptides (▪ for GCRP; • for chGIP; ◊ for chGLP2; ○ for chGCG; △ for huGLP1; ▾ for Exe-4). Species-specific responses of chGCRPR (D), xeGCRPR (E), and mdGCRPR (F) were determined by treating cells with increasing concentrations of GCRP (▾ for chGCRP; ? for xeGCRP; • for mdGCRP) for 6 h, and luciferase activity was examined.

To identify authentic ligands for GCRPR, we cloned GCRPR cDNAs from chicken (ch), *Xenopus* (xe), and medaka (md). Each *GCRPR*-expressing plasmid was co-transfected into HEK293T cells with the pCRE-luc reporter gene to examine Gα_s_-mediated signal activation [49]. Cells were then treated with increasing concentrations of various peptide forms, including GCRP, GIP, GLP1, GLP2, GCG, and exendin-4 (Table 1). GCRP from each species induced a concentration-dependent increase in luciferase activity with the highest potency toward its cognate receptor (EC_50_∶0.87 nM chGCRP, 2.24 nM xeGCRP, and 6.76 nM mdGCRP). Related peptides exhibited relatively low potencies toward GCRPR (Table 2 and Figs. 3A, B, and C). It is of particular interest to note that the potency of exendin-4 at chGCRPR and xeGCRPR was clearly lower than GCRP, whereas the potency at mdGCRPR was very similar to that of mdGCRP (Fig. 3C).

**Table 1:** Amino acid sequences for GCRPs and related peptides.

Peptides	1	11	21	31	41
Chicken GCRP	HSEGTFTSDF	TRYLDKMKAK	DFVHWLINT		
Xenopus GCRP	HSEGTFSSDL	TRYLDKMKAK	DFVQWLMN		
Medaka GCRP	HTDGMFTSDL	TNYLDKMKAK	NFVEWLAAIK	QQE	
Human GLP1	HAEGTFTSDV	SSYLEGQAAK	EFIAWLVKGR		
Chicken GLP1	HAEGTYTSDI	TSYLEGQAAK	EFIAWLVNGRa		
Medaka GLP1	HADGTFTSDV	SAYLKEQAIK	DFVAKLKSGQ	I	
Human GLP2	HADGSFSDEM	NTILDNLAAR	DFINWLIQTK	ITD	
Chicken GLP2	HADGTFTSDI	NKILDDMAAK	EFLKWLINTK	VTQ	
Medaka GLP2	HVDGSFTSDV	NKVLDSMAAK	EYLLWVMTSK	PSNE	
Human GIP	YAEGTFISDY	SIAMDKIHQQ	DFVNWLLAQK	GKKNDWKHNI	TQ
Chicken GIP	YSEATLASDY	SRTMDNMLKK	NFVEWLLARR	EKKSDNVIEP	Y
Human GCG	HSQGTFTSDY	SKYLDSRRAQ	DFVQWLMNT		
Chicken GCG	HSQGTFTSDY	SKYLDSRRAQ	DFVQWLMST		
Medaka GCG1	HSEGTFSNDY	SKYLEDRKAQ	DFVRWLMNN		
Medaka GCG2	HSEGTFSNDY	SKYLETRRAH	DFVQWLKNS		
Exe-4	HGEGTFTSDL	SKQMEEEAVR	LFIEWLKNGG	PSSGAPPPSG	
Chicken [GQA]GCRP	HSEGTFTSDF	TRYLDGQAAK	DFVHWLINT		
Chicken [KMK]GLP1	HAEGTFTSDV	SSYLEKMKAK	EFIAWLVKGR		

**Table 2:** Ligand potency (EC_50_ value) and efficacy (E_max_ value) for GCRPR.

	chGCRPR		xeGCRPR		mdGCRPR	
peptides	EC_50_	E_max_	EC_50_	E_max_	EC_50_	E_max_
	[nM]	[fold induction]	[nM]	[fold induction]	[nM]	[fold induction]
chGCRP	0.87±0.13	6.00±0.09	5.86±1.11	30.09±0.72	33.73±4.64	8.12±0.17
xeGCRP	1.18±0.46	6.25±0.23	2.24±0.30	30.43±0.49	30.62±6.93	8.10±0.27
mdGCRP	68.23±17.28	6.93±0.22	94.62±12.87	31.96±0.75	6.84±1.07	7.90±0.16
Exe-4	14.45±2.63	6.00±0.15	26.24±3.11	31.52±0.56	9.06±6.66	8.04±0.20
chGIP	69.18±10.66	6.22±0.16	274.16±62.20	32.35±1.53	ND	ND
huGLP1	119.95±27.06	6.24±0.25	390.84±69.39	35.00±1.39	ND	ND
chGLP2	33.42±7.06	6.26±0.19	ND	ND	ND	ND
mdGLP1	ND	ND	ND	ND	NR	NR
mdGLP2	ND	ND	ND	ND	NR	NR
huGCG	35.32±7.95	6.30±0.21	49.43±8.38	32.73±0.96	ND	ND
mdGCG1	ND	ND	ND	ND	107.89±33.20	4.26±0.19
mdGCG2	ND	ND	ND	ND	NR	NR

Results are presented as mean ± S.E. of at least three independent experiments. NR, no response; ND, not determined.

Although GCRPs exhibit a high degree of sequence similarity with one another, there are some variations in amino acid sequences. These differences might cause species-specific activity of GCRP toward GCRPR. GCRPs from chicken, *Xenopus*, and medaka were added to cells expressing GCRPR from different species (Table 2 and Figs. 3D, E, and F). Chicken and *Xenopus* GCRPs revealed similarly high potencies for chGCRPR and xeGCRPR, whereas mdGCRP exhibited significantly lower potencies for these receptors. All three peptides showed similar potencies toward mdGCRPR (Fig. 3F). The low potency of mdGCRP toward chGCRPR and xeGCRPR may be due to variations in the amino acid sequences, particularly at positions 3, 5, 12, 21, and 24.

We then examined whether GCRP can activate other GCRPR-related receptors such as GLP1R, GCGR, and GIPR in chicken, *Xenopus*, and medaka. The results revealed that GCRP barely activated the GCRPR-related receptors in these species (data not shown). Together, these results suggest that GCRP is likely an authentic ligand for GCRPR at least in terms of their pharmacological properties.

### Human Receptors for Glucagon Family Peptides Respond Weakly to GCRPs

**Figure 4:**
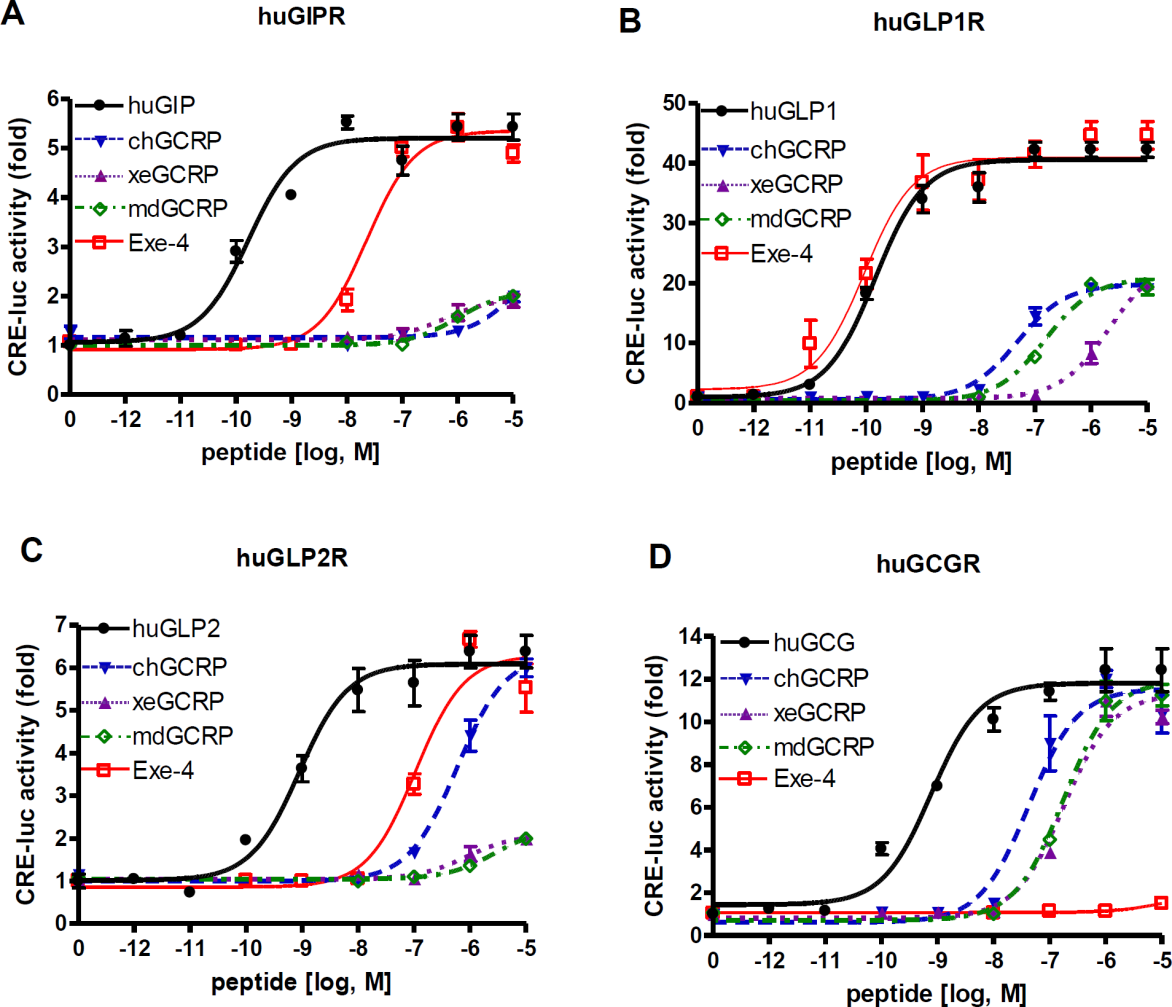
GCRP activities toward the human GCGR family. Plasmids containing huGIPR (A), huGLP1R (B), huGLP2R (C), or huGCGR (D) cDNA were co-transfected with the CRE-luc reporter vector into HEK293T cells. Forty-eight hours after transfection, cells were treated with various concentrations of human peptides (• for huGIP, huGLP1, huGLP2, and huGCG), GCRP (▾ for chGCRP; ▴ for xeGCRP; ◊ for mdGCRP), and Exe-4 (□) for 6 h. Luciferase activity was then determined.

Exendin-4 exhibited a very strong potency toward mammalian GLP1R. Thus, we examined the potency of GCRP to human GLP1R, GCGR, GLP2R, and GIPR in comparison with exendin-4. HEK293T cells transfected with human *GIPR*, *GLP1R*, *GLP2R*, or *GCGR* were treated with GCRPs from three different species, exendin-4, and their corresponding ligand forms (Table 1). All receptors were activated by their cognate ligands with the highest potencies. Exendin-4 fully activated huGLP1R with a potency similar to GLP1 (EC_50_≈0.1 nM) and activated huGIPR and huGLP2R with moderate potency (EC_50_≈0.1 µM) but not huGCGR. In contrast to exendin-4, GCRPs activated huGLP1R, huGLP2R, and huGCGR only at high concentrations. GCRPs marginally activated huGIPR even at 10 µM. (Table 3 and Fig. 4). This result suggests that GCRPs exhibit pharmacological activity distinct from that of exendin-4.

**Table 3:** Ligand potency (EC_50_ value) and efficacy (E_max_ value) for the human GCGR family.

	huGIPR		huGLP1R		huGLP2R		huGCGR	
peptides	EC_50_	E_max_	EC_50_	E_max_	EC_50_	E_max_	EC_50_	E_max_
	[nM]	[foldinduction]	[nM]	[foldinduction]	[nM]	[foldinduction]	[nM]	[foldinduction]
Exe-4	22.86±5.61	5.36±0.15	0.09±0.03	40.95±1.51	107.15±31.35	6.29±0.28	NR	NR
chGCRP	NR	3.03±2.41	48.87±9.15	19.84±0.57	591.56±126.74	6.32±0.24	42.66±14.47	6.32±0.24
xeGCRP	NR	1.95±0.14	>1000	24.11±1.68	NR	NR	181.55±39.49	2.09±0.12
mdGCRP	NR	2.11±0.09	155.96±32.84	20.90±0.74	NR	NR	175.39±41.78	2.27±0.11
huGIP	0.17±0.05	5.21±0.12	ND	ND	ND	ND	ND	ND
huGLP1	ND	ND	0.14±0.03	40.61±0.73	ND	ND	ND	ND
huGLP2	ND	ND	ND	ND	0.89±0.25	6.09±0.09	ND	ND
huGCG	ND	ND	ND	ND	ND	ND	0.78±0.21	6.09±0.18

Results are presented as mean ± S.E. of at least three independent experiments. NR, no response; ND, not determined.

### Receptor-binding Affinity of GCRP

**Figure 5:**
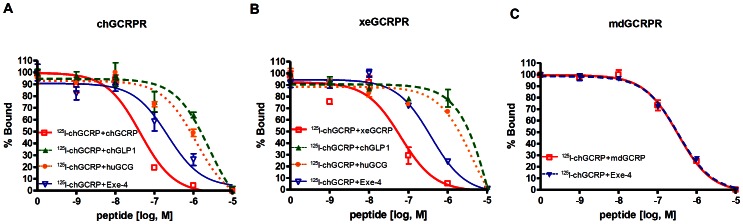
Ligand-binding affinity of GCRP receptors. A cell-binding assay was performed in HEK293T cells expressing chGCRPR (A), xeGCRPR (B), or mdGCRPR (C) with ^125^I-chGCRP in the presence of various concentrations of cold ligand (□ for GCRP; ▴ for chGLP1; • for huGCG; ▽ for Exe-4).

To confirm the binding affinities of GCRPs to their receptors, we adopted a competitive displacement binding assay. Because chGCRP exhibited high potencies toward ch-, xe-, and mdGCRPR in the reporter assay system (Fig. 3), we labeled chGCRP with ^125^I. HEK293T cells transfected with each GCRPR were incubated with ^125^I-chGCRP in the absence or presence of increasing concentrations of various unlabeled cold peptides. A displacement binding assay revealed that each GCRP had the highest binding affinity for its cognate receptor, which is consistent with the results obtained from the reporter assay (Fig. 3). The IC_50_ values were 43.65 nM for chGCRP, 63 nM for xeGCRP, and 0.32 µM for mdGCRP (Table 4 and Fig. 5). Exendin-4 exhibited relatively higher affinity for chGCRPR and xeGCRPR in comparison to related peptides. Interestingly, the affinity of exendin-4 for mdGCRPR is similar to that of mdGCRP, indicating that exendin-4 retains the ability to bind and activate mdGCRPR (Fig. 5).

**Table 4:** Ligand affinity (IC_50_) toward GCRPRs.

	IC_50_ [nM]		
peptides	chGCRPR	xeGCRPR	mdGCRPR
GCRP	43.55±11.11	65.53±20.44	327.34±51.02
chGLP1	>1000	>1000	ND
huGCG	>1000	>1000	ND
Exe-4	217.77±93.19	374.11±84.88	337.29±42.82

Results are presented as mean ± S.E. of at least three independent experiments. ND, not determined.

### Intracellular GCRPR Signaling

**Figure 6:**
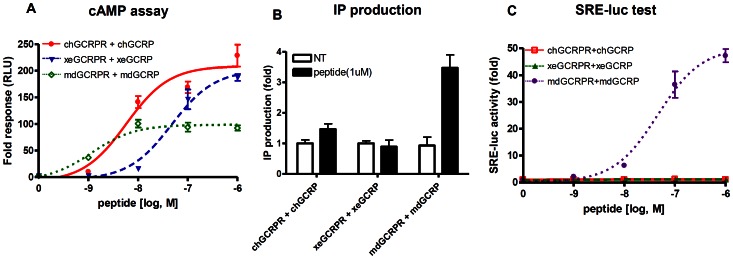
GCRPR-mediated signaling pathway. cAMP accumulation (A) or IP production (B) in response to various concentrations of GCRP was determined in HEK293T cells transfected with plasmids containing GCRP receptors (• for chGCRPR; ▾ for xeGCRPR; ◊ for mdGCRPR). mdGCRP induces SRE-luc activity through mdGCRPR (C). Forty-eight hours after transfection with the receptor and the reporter genes, HEK293T cells were treated with different concentrations of GCRPs for 6 h. Luciferase activity was determined.

Upon ligand binding, GCGR family receptors induce Gα_s_-mediated adenylyl cyclase activity and increase intracellular cAMP production [6], [50]. Thus, we hypothesize that GCRPRs may also stimulate this pathway to generate cAMP. Indeed, our reporter assay demonstrated that activation of GCRPR induced cAMP responsive element (CRE)-driven reporter activity (Fig. 3). To further confirm the intracellular signaling pathway of GCRPRs, we measured cAMP levels by real-time luminescence in cells expressing *Glosensor-22F cAMP*, which interacts directly with cAMP [51]. As shown in Fig. 6A, GCRPs increase cAMP levels by stimulating their cognate receptors with high potencies (EC_50_, chGCRP: 5.8 nM; xeGCRP: 46 nM; mdGCRP: 1.15 nM), suggesting that GCRPR activates the Gα_s_–mediated signaling pathway (Fig. 6A). Because some GCGR family members stimulate intracellular calcium accumulation in a certain condition [52], it can be postulated that GCRPR activates the Gα_q/11_-PLCβ pathway. Thus, we measured GCRP-induced accumulation of inositol phosphates (IP) in HEK293T cells transfected with the *GCRPR* genes. The mdGCRP induced IP production through its cognate receptor, whereas neither chGCRPR nor xeGCRPR were able to increase IP levels (Fig. 6B). The ability of mdGCRPR to stimulate Gα_q/11_-mediated signaling was further determined with a SRE-luc reporter assay system [43], [49], [53]. The SRE-luc reporter assay revealed that only mdGCRPR but not ch- and xe-GCRPR stimulated SRE-driven transcription activity (Fig. 6C). These data suggest that mdGCRPR acquired the ability to activate Gα_q/11_-mediated signaling in addition to Gα_s_-mediated signaling. Alternatively, chGCRPR and xeGCRPR may have evolutionarily lost the ability to stimulate Gα_q/11_-mediated signaling.

### Partial Identification of the Motif Conferring Ligand Specificity toward GCRPR

**Figure 7:**
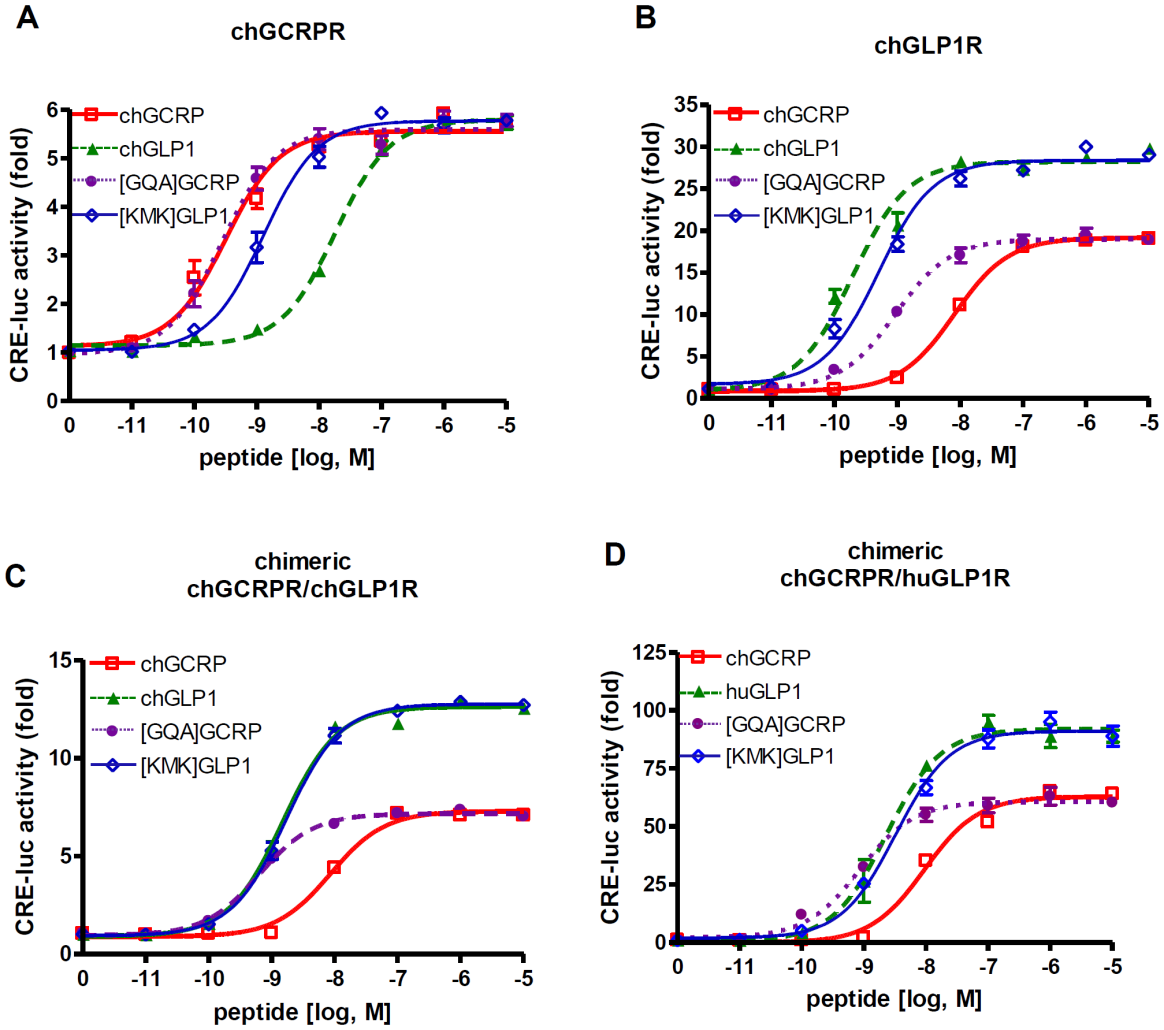
Potencies and efficacies of chimeric GCRP/GLP1 peptides toward chimeric GCRPR/GLP1R. Plasmids containing wild-type chGCRPR (A), chGLP1R (B), chimeric chGCRPR/chGLP1R (C), or chimeric chGCRPR/huGLP1R (D) cDNAs were introduced into HEK293T cells with the CRE-luc plasmid. Luciferase activities stimulated by graded concentrations of chGCRP (□), chGLP1 (▴), ch[GQA]GCRP (•), or ch[KMK]GLP1 (◊) were determined.

Although GCG family peptide sequences are relatively similar, each specifically activates its cognate receptor with a sub-nanomolar EC_50_ value in most cases. This implies that a unique motif in each peptide mediates interaction with the receptor. To identify GCRP-specific amino acids, we compared its sequence with other GCG family members. We found that residues at positions 16–18 exhibited peptide-specific modifications such that the K^16^M/I^17^K^18^, S/T^16^R^17^R^18^, and G/D^16^Q^17^A^18^ motifs were conserved in GCRP, GCG, and GLP1, respectively. This observation raises the possibility that the K^16^M/I^17^K^18^ motif in GCRP allows the peptide to distinguish GCRPR from other GCG family receptors. To examine the effect of this region on receptor activation, we made mutant peptides of chicken GCRP and GLP1 in which the regions with G^16^Q^17^A^18^ and K^16^M^17^K^18^ were swapped. We compared the activities of each mutant peptide on each receptor. Wild-type GCRP and [G^16^Q^17^A^18^]GCRP activated chGCRPR with similar potency. However, [K^16^M^17^K^18^]GLP1 was significantly more potent toward chGCRPR than wild-type GLP1 but similar to GCRP (Table 5 and Fig. 7A). Similarly, GLP1 and [K^16^M^17^K^18^]GLP1 activated chGLP1R with similar potencies and efficacies, whereas [G^16^Q^17^A^18^]GCRP was more potent than GCRP (Table 5 and Fig. 7B). Thus, replacement of K^16^M^17^K^18^ in GLP1 and G^16^Q^17^A^18^ in GCRP greatly enhanced potency toward GCRPR and GLP1R, respectively, indicating that these residues are potentially important for determining receptor specificity. However, swapping motifs did not significantly change the potencies and efficacies of peptides toward their cognate receptors, suggesting that other regions in these peptides may compensate for the role of the mutated residues.

**Table 5:** Ligand potency (EC_50_ value) and efficacy (E_max_ value) for GCRPR, GLP1R, and chimeric receptors.

	chGCRPR		chGLP1R		chimeric chGCRPR/chGLP1R		chimeric chGCRPR/huGLP1R	
peptides	EC_50_	E_max_	EC_50_	E_max_	EC_50_	E_max_	EC_50_	E_max_
	[nM]	[foldinduction]	[nM]	[foldinduction]	[nM]	[foldinduction]	[nM]	[foldinduction]
chGCRP	0.32±0.06	5.56±0.09	8.03±0.60	19.16±0.19	8.57±0.87	7.30±0.09	9.44±1.31	62.88±1.27
chGLP1	18.28±1.76^a^	5.80±0.07	0.20±0.03^a^	28.23±0.39^a^	1.52±0.13^a^	12.64±0.13^a^	2.29±0.44^a^	91.94±2.18^a^
[GQA]GCRP	0.28±0.05^b^	5.60±0.09	0.94±0.13^a^	18.99±0.30^b^	0.63±0.06^a^	7.18±0.07^b^	0.86±0.14^a^	60.62±1.15^b^
[KMK]GLP1	1.02±0.19^b^	5.77±0.10	0.50±0.06^a^	28.38±0.39^a^	1.71±0.14^a^	12.77±0.12^a^	3.25±0.48^a^	91.15±1.65^a^

Results are presented as mean ± S.E. of at least three independent experiments. a, P<0.05 *vs.* chGCRP; b, P<0.05 *vs.* chGLP1.

Biochemical data and structural analysis of the ligand-bound ECD of the receptor suggested that the N-termini of GCG peptides bind to core regions of the receptors, whereas the second half of the α-helix of the peptide interacts with the ECD of the receptor. Interestingly, G^16^Q^17^A^18^ in GLP1 and K^16^M^17^K^18^ in GCRP reside at the border between regions that interact with the core domain and ECD. To explore the regions of the receptor that interact with K^16^M^17^K^18^ or G^16^Q^17^A^18^, we designed a chimeric receptor containing the N-terminal ECD of GCRPR and the core domain of GLP1R (GCRPR/GLP1R) (Figs. 7 C and D). GLP1 and [K^16^M^17^K^18^]GLP1 exhibited higher potencies and efficacies than GCRP toward the chimeric receptors. Interestingly, introducing G^16^Q^17^A^18^ into GCRP significantly increased potency but not efficacy toward the chimeric receptors (Table 5 and Figs. 7 C and D). The potencies and efficacies of the peptides toward chimeric receptors containing the core domain of GLP1R are similar to those toward wild-type GLP1R. Thus, it can be postulated that the K^16^M^17^K^18^ and G^16^Q^17^A^18^ motifs are likely to interact with the core domain of the receptor. We have constructed chimeric receptors containing the GCRPR core domain with human or chicken GLP1R ECDs. However, all these constructs responded very poorly to the ligands including GLP1, GCRP, and chimeric peptides (Data not shown). Probably, these combinations of the GLP1R ECD and the GCRPR core domain may induce unexpected conformation which does not allow either ligand binding or receptor activation.

## Discussion

The GCG-related peptide and receptor family is one of the best-studied ligand and receptor families, particularly in gut-brain interaction systems. In the current study, we used bioinformatics to identify GCRP and GCRPR, which are novel members of this ligand-receptor family. These new members are present in a variety of tetrapods and teleost but not in mammals and zebrafish. Genome synteny and phylogenetic analyses revealed that GCRP is likely to have emerged through 2R. Thus, GCRP, GCG, and GIP are ohnologous to one another. Two *GCRP* genes in chicken may have evolved through local duplication following 2R during the emergence of birds. In contrast, the evolutionary history of GCRPR is more complex. Genome synteny analyses suggested that GCRPR, GCGR, GLP2R, and GLP1R were on a common genome fragment that was a paralogon of GIPR-containing genome fragments. This observation indicates that GCRPR, GCGR, GLP2R, and GLP1R have emerged through local duplication following 2R before the divergence of tetrapods and teleosts [40]. According to our phylogenetic and synteny analyses, many teleost species with the exception of zebrafish have retained *GCRPR* but lost *GIPR*. Similarly, teleost species that express *GCRP* do not have the gene encoding the GIP peptide. Thus, with the exception of zebrafish, teleost species may have lost the *GIP* and *GIPR* system following 2R before the divergence of teleost and tetrapods.

Prior to our study, GCRP was classified as a member of the exendin-4 family [46]. However, our phylogenetic study revealed that GCRP is more closely related to GIP versus other peptide members, whereas exendin-4 is more similar to GLP1 with respect to its amino acid sequence. In addition, GCRP has biochemical properties distinct from those of exendin-4, GCG, GLP1, GLP2, and GIP. Chicken, *Xenopus*, and medaka GCRPs exhibited the highest potencies and affinities toward their corresponding GCRPRs but showed very low potencies to human GIPR, GLP1R, GLP2R, and GCGR. Interestingly, exendin-4 showed a similar potency and affinity for medaka GCRPR as medaka GCRP but much greater potency for human GLP1R than medaka GCRP. This unexpected result may be due to the promiscuous activity of exendin-4. Indeed, exendin-4 can fully activate GLP1R and moderately activates GIPR, GLP2R, and GCRPR. Our biochemical observations clearly indicate that GCRP is a functional ligand for GCRPR.

Most mammalian GCGR family members are coupled primarily to the Gα_s_-mediated signaling pathway [5]. Although signaling downstream of non-mammalian GCGR is not fully determined, this receptor family is considered to be coupled to Gα_s_. Some family members can induce calcium accumulation and other signaling pathways [52]. However, these responses may be secondary effects related to increased intracellular cAMP [54]. Similarly, GCRPR is coupled primarily to Gα_s_, as demonstrated by increased cAMP levels and CRE-luc reporter activity in response to GCRP stimulation. Interestingly, medaka GCRPR but not *Xenopus* or chicken GCRPR activated Gα_q/11_-mediated signaling. Stimulation of medaka GCRPR increased cytoplasmic IP levels and SRE-luc reporter activity. Thus, receptor coupling to both the Gα_s_ and the Gα_q/11_ pathways is a unique characteristic of medaka GCRPR. However, the physiological relevance of this dual coupling remains to be investigated.

Duplicated paralogs of peptides and receptors have undergone sequence modifications during evolution, leading to diversification and specification of ligand-receptor pairs. Specific diversification of peptides, in other words, conservation within orthologs but variation among paralogs could confer selective interaction of a peptide with the cognate receptor, allowing discrimination of paralogous receptors [27], [55], [56], [57]. Consistent with this hypothesis, we observed that the K^16^M/I^17^K^18^ motif in GCRP is conserved amongst vertebrates but shows variation from the corresponding residues in other peptides. These positions are relatively conserved for GCG (S/T^16^R^17^R^18^) and GLP1 (G/D^16^Q^17^A^18^), indicating that the motif at positions 16–18 contributes to selective interaction with the cognate receptor. Indeed, chimeric [K^16^M^17^K^18^]GLP1 exhibited increased potency toward chGCRPR compared to wild-type chicken GLP1. Chimeric [G^16^Q^17^A^18^]GCRP was more potent for chGLP1R than wild-type chicken GCRP, suggesting that these motifs, at least in part, contribute to selective recognition of their cognate receptors. However, it is unclear why GCRP with G^16^Q^17^A^18^ and GLP1 with K^16^M^17^K^18^ retained wild-type-like potencies to GCRPR and GLP1R, respectively. It is likely that modification of these residues can be structurally compensated, allowing high potency to the corresponding receptors to be retained.

In contrast to the motif at positions 16–18, GCRP exhibited high conservation of residues that are known to be important for receptor interaction. For example, residues Ala^19^, Lys^20^, Phe^22^, Ile^23^, Leu^26^, and Val^27^ in the α-helix of GLP1 directly contact the ECD of GLP1R [24]. Ala^19^, Lys^20^, Phe^22^, and Leu^26^ are identical to residues in the corresponding positions of GCRP. Further, Ile^23^ and Val^27^ are replaced by Val^23^ and Ile^27^ in GCRP, which have similar biochemical properties to Ile^23^ and Val^27^, respectively. His^1^, Gly^4^, Phe^6^, Thr^7^, and Asp^9^ of GLP1 are critical for maintaining the secondary structure and for mediating interaction with the receptor [27], [31], [34]. These residues in GLP1 are identical to those of GCRP, which may confer cross-reactivity of these peptides with related receptors. Further, this similarity may lead to high and full potency of [G^16^Q^17^A^18^]GCRP toward GLP1R and chimeric GCRPR/GLP1R, respectively.

The residues in the receptor core domain and ECD that mediate interaction with the K^16^M^17^K^18^/G^16^Q^17^A^18^ motif of peptides remain unknown. The crystal structure of the GLP1-bound ECD of GLP1R demonstrated that amino acid residues between Ala^18^ and Val^27^ of the peptide bind to the ECD of the receptor [24]. Similarly, other crystal structures of ligand-bound ECDs suggested that residues starting from position 18 or 19 interact with the ECD of the receptor [28]. Thus, motifs at positions 16–18 of the peptides are at the border between residues that interact with the ECD and residues that contact the core domain. Particularly, Gly^16^ of GLP1 is involved in the formation of a kink in the peptide α-helix [24]. Our study showed that the GLP1R and GCRPR/GLP1R chimeric receptor, both of which contain the GLP1R core domain, responded better to GLP1 than GCRP. Further, [G^16^Q^17^A^18^]GCPR increased potency to this chimeric receptor up to that of wild-type GLP1, indicating that the G^16^Q^17^A^18^ motif may contact the core domain of the chimeric receptor. Thus, the K^16^M^17^K^18^/G^16^Q^17^A^18^ motif in the peptide may interact with residues in the core domain of the receptor.

In conclusion, our discovery of GCRP and its receptor demonstrates the evolutionary history of GCG-related peptides and their receptors in vertebrates. Duplication of the ancestral genes encoding these peptide and receptor families followed by specific modifications in the amino acid sequences of GCG family peptides and receptors contributes to selective interaction of a ligand with its corresponding receptor. Further identification of specific residues in GCRP, GLP1, and their receptors is particularly important for determining the structure of ligand binding pocket in the receptor. This information will facilitate the development of potent agonists and antagonists of GCGR family members, which are involved in a variety of pathophysiological processes.

## Materials and Methods

### Data Retrieval and Phylogenetic Analysis of GCG Family Peptides and Receptors

The amino acid sequences of GCG-like family peptides and receptors were downloaded from either the Ensembl Genome Browser (http://www.ensembl.org) or the GenBank database with the Entrez data retrieval tool (http://www.ncbi.nlm.nih.gov/Entrez/). Specifically, search tools provided by Ensembl Genome Browser for orthologous or paralogous genes identified genes from non-mammalian vertebrates. This data set was the start set. Genes that were not part of the start set were manually searched against the human, mouse, chicken, anole lizard, *Xenopus tropicalis*, zebrafish, medaka, fugu, stickleback, and tetraodon genome databases with the TBLASTN algorithm. Putative signal peptides were predicted by SignalP 3.0 (http://www.cbs.dtu.dk/services/SignalP/). The full set of GCG-like peptide and receptor sequences were aligned on the Windows version of ClustalX-2.1 (Gonnet matrix, gap opening penalty 10, and gap extension 0.2). Alignments were bootstrapped 1000 times, and neighbor-joining trees were obtained using MEGA 5.05.

### Chemicals and Hormones

All chemicals were obtained from Sigma-Aldrich (St. Louis, MO, USA) unless otherwise stated. Restriction enzymes were obtained from New England Biolabs (Ipswich, MA, USA). Wild-type human (hu) GIP, huGLP1, huGLP2, huGCG, chicken (ch) GCRP, chGLP1, chGIP, chGLP2, *Xenopus* (xe) GCRP, medaka (md) GCRP, mdGLP1, mdGLP2, mdGCG1, mdGCG2, chimeric [KMK]GLP1, and chimeric [GQA]GCRP were synthesized by AnyGen (Gwangju, Korea). All peptide sequences are listed in Table 1. The purities of the synthesized peptides were greater than 98% according to high-performance liquid chromatography. All peptides were dissolved in DMSO and then diluted in media to the desired working concentrations.

### RNA Isolation and Reverse Transcriptase-polymerase Chain Reaction (RT-PCR)

Brain, liver, and pancreatic tissues collected from White Leghorn hen were provided by Biopoa Co Ltd. (Suwon, Korea). Animal procedures were approved by the institutional animal care and use committee (IACUC) of the Biopoa Co. Ltd. (Permit number: BP-2013–0008). All efforts were made to minimize suffering. Total RNA was extracted from frozen tissues with TRI Reagent (Molecular Research Center Inc., Cincinnati, OH, USA) according to the manufacturer’s instructions. First-strand cDNAs were prepared by incubating total RNA with a random hexamer and mouse mammary tumor virus reverse transcriptase (Promega Corp., Madison, WI, USA) at 37°C for 1 h. *Xenopus* cDNAs were obtained as previously described [43]. To clone full-length open-reading frame cDNAs for GCRPRs, gene-specific primers were designed based on the predicted sequences retrieved from the Ensembl Genome Database. PCR conditions were as follows: denaturation at 95°C for 5 min, followed by different cycles at 95°C for 1 min, 58°C for 1 min, and 72°C for 1 min.

### Plasmid Constructs

The pcDNA3.1 expression vector was purchased from Invitrogen Corp. (San Diego, CA, USA). The CRE-luc vector, which contains four copies of the cyclic AMP-responsive element (CRE; TGACGTCA), was purchased from Stratagene (La Jolla, CA, USA). The cDNA for huGIPR was kindly provided by Dr. Bernard Thorens (Institute of Pharmacology and Toxicology, Switzerland). The cDNAs for huGLP2R and huGCGR were from BRN SCIENCE Inc. (Seoul, Korea). The cDNA for medaka GCRPR was synthesized by GenScript (Piscataway, NJ, USA). The cDNAs for chGCRPR and xeGCRPR were obtained by RT-PCR from chicken and *Xenopus* tissues. All genes were constructed at the *EcoR*I and *Xho*I sites of pcDNA3.1 by PCR using appropriate primers from CosmogenTech Inc. (Seoul, Korea). The identity of each gene was verified by sequencing.

Chimeric forms of chGCRPR and GLP1R were generated by standard overlap-extension PCR with an appropriate primer set [27]. The N-terminal extracellular region covering 145 amino acids of chGCRPR was fused with the core domain of chGLP1R or huGLP1R ranging from the first transmembrane region (starting from amino acid position 144 for chGLP1R and position 148 for huGLP1R) to the C-terminal intracellular region. The chimeric gene was inserted into the *EcoR*I and *Xho*I sites of pcDNA3.1.

### Cell Transfection and Luciferase Assay

HEK293T cells (American Type Culture Collection, Manassas, VA, USA) were maintained in Dulbecco’s modified Eagle’s medium (DMEM) supplemented with 10% fetal bovine serum, 100 U/ml penicillin G, and 100 µg/ml streptomycin (Invitrogen). Cells were seeded in 48-well plates at a density of 1.4×10^4^ cells per well 1 day before transfection. A mixture containing 75 ng of pGL3-CRE-luciferase reporter construct, 75 ng of expression plasmid, and 2 µl of Effectene reagent (Qiagen, Chatsworth, CA, USA) was prepared and added to each well according to the manufacturer’s instructions. Approximately 48 h after transfection, cells were treated with the respective ligands for 6 h. Cells were lysed by adding 100 µl lysis buffer and the luciferase activity in 40 µl of cell extract was determined on a luciferase assay system according to the standard protocol for the Synergy 2 Multi-Mode Microplate Reader (BioTek, Winooski, VT, USA).

### Measurement of Inositol Phosphate Production

An IP production assay was performed as described previously [53]. HEK293T cells were seeded into a 12-well plate and transfected with the chGCRPR, xeGCRPR, or mdGCRPR plasmid. After transfection, cells were incubated in M199 medium (Invitrogen) in the presence of 1 µCi/ml *myo*-[^3^H] inositol (Amersham Biosciences, Piscataway, NJ, USA)/well for 20 h. Media were removed, and cells were washed with 0.5 ml Buffer A (140 mM NaCl, 20 mM HEPES, 4 mM KCl, 8 mM D-glucose, 1 mM MgCl2, 1 mM CaCl2, and 1 mg/ml fatty acid-free bovine serum albumin). Cells were then preincubated for 30 min with Buffer A containing 10 mM LiCl. The reaction was terminated by replacing incubation media with 0.5 ml ice-cold 10 mM formic acid. After 30 min at 4°C, formic acid extracts were transferred to columns containing Dowex anion-exchange resin (AG-1-X8 resin, Bio-Rad). Total IPs were then eluted with 1 ml ammonium formate and 0.1 M formic acid. Radioactivity was measured on a Tri-Carb 3100TR scintillation counter (PerkinElmer Life Sciences, Waltham, MA, USA).

### Binding Assay

chGCRP was radioiodinated by the chloramine-T method and then purified by chromatography on a Sephadex G-25 column (Sigma-Aldrich) in 0.01 M acetic acid and 0.1% BSA [27]. HEK293T cells were transfected with GCRPR (300 ng of DNA/well in 12-well plates) with Effectene (Qiagen). Cells were washed after 48 h and incubated for one additional hour with binding buffer (serum-free DMEM with 0.1% BSA, pH 7.4) containing 100,000 cpm ^125^I-labeled ligand in the absence or presence of various concentrations of cold peptides. Cells were washed twice with ice-cold Dulbecco’s PBS and dissolved in 1% SDS and 0.2 M NaOH. Radioactivity was measured with the Wallac 1489 Wizard 3 γ-counter (PerkinElmer Life Science).

### cAMP Accumulation

GCRP-induced cAMP mobilization was measured in HEK293T cells that stably express the pGlosensor™-22F cAMP plasmid (Promega Corp., Madison, WI, USA). Glosensor-22F cAMP HEK293T cells were seeded into 96-well plates 24 h before transfection. After 48 h, Glosensor cAMP substrates were added to cells in CO_2_-independent media. After 2 h, cells were stimulated, and luminescence was measured for up to 30 min at 26°C in a microplate reader.

### Data Analysis

Data analysis was performed by nonlinear regression with a sigmoidal dose-response. The agonist concentrations that induced half-maximal stimulation (EC_50_) or half-maximal inhibition of binding (IC_50_) were calculated with GraphPad PRISM4 software (GraphPad Software Inc., San Diego, CA, USA). All data were presented as mean ± S.E. of at least three independent experiments. Group means were compared by Student’s *t* test or one-way analysis of variance followed by Bonferroni’s multiple comparison tests. *p*<0.05 was accepted as significant.

## Supporting Information

Figure S1 **Synteny for the GCRP family gene-containing chromosomes of vertebrates**. ***A,*** The genomic locations of the *GCRP* gene and its neighboring genes were compared with those of their orthologous genes in vertebrates, including human, anole, chicken, *Xenopus*, zebrafish, medaka, tetraodon, and stickleback. Orthologous genes are aligned in the same column. Chromosome numbers are labeled above the indicated gene. Chromosomal locations (megabases) are shown beneath the gene. The genome scaffold numbers for *Xenopus* started with the same GL17 followed by four variable digits. Only the variable numbers are shown above the gene. For example, if a scaffold number for *Xenopus GCRP* is GL172703, only 2703 is written above the gene in the figure. Similarly, the last four digits of the genome scaffold numbers for anole are shown. If fish genes were doubled due to teleost-specific genome duplication, the chromosome numbers and gene locations of the doubled genes are indicated in two lines with different colors. The absence of the peptide genes is indicated by white boxes with broken lines, below which ‘x’ is labeled. ***B,*** Four putative early paralogons containing the GCRP family peptide gene with their neighboring genes. Human chromosome numbers are above the indicated gene, and the gene locations (megabase) are shown beneath the gene. Paralogs of each gene in different paralogons were aligned on the same column with the same colors. The positions of each gene block in the gnathostome ancestor chromosome (*GAC*) in the N-models are indicated in the left.
(PDF)

Figure S2 **Synteny for the chromosomal locations of GCRPR family genes in vertebrates.** ***A,*** The genome locations of *GCRPR*, *GCGR*, *GLP2R*, *GLP1R*, *GIPR*, and their neighboring genes in vertebrates, including human, anole, chicken, *Xenopus*, zebrafish, medaka, tetraodon, and stickleback are shown. Orthologous genes are aligned on the same column. Chromosome numbers are shown above the indicated gene, and the gene locations (megabase) are shown beneath the gene. The absence of the receptor genes is indicated by white boxes with broken lines, below which ‘x’ is labeled. The positions of each gene block in the gnathostome ancestor chromosome (*GAC*) in the N-models are indicated at the top of the column. ***B,*** Local duplication of GCGR, GLP2R, GCGPR, and GLP1R after two rounds of whole genome duplication. GIPR and its neighboring genes are aligned on human chromosome 19 of *GAC* G2. Although GCGR, CCRPR, GLP2R, and GLP1R are on different *GAC* blocks, they are aligned on one paralogon based on synteny with GIPR-containing paralogon. Two additional paralogons missing GCGR family members were identified by synteny analyses. Human chromosome numbers are shown above the indicated gene, and the gene locations (megabase) are shown beneath the gene. Ohnologs of each gene on different paralogons were aligned on the same column with the same colors and connected by straight lines. Paralogs were connected by a dashed line. Local duplications were indicated by curved lines.
(PDF)

Information S1 (PDF)
