# Supplementary figures and images for: A Novel Glucagon-Related Peptide (GCRP) and Its Receptor GCRPR Account for Coevolution of Their Family Members in Vertebrates
Source: PLoS One. 2013 Jun 11;8(6):e65420. doi: 10.1371/journal.pone.0065420 (PMC3679108; doi:10.1371/journal.pone.0065420)

Supplementary Figure S1

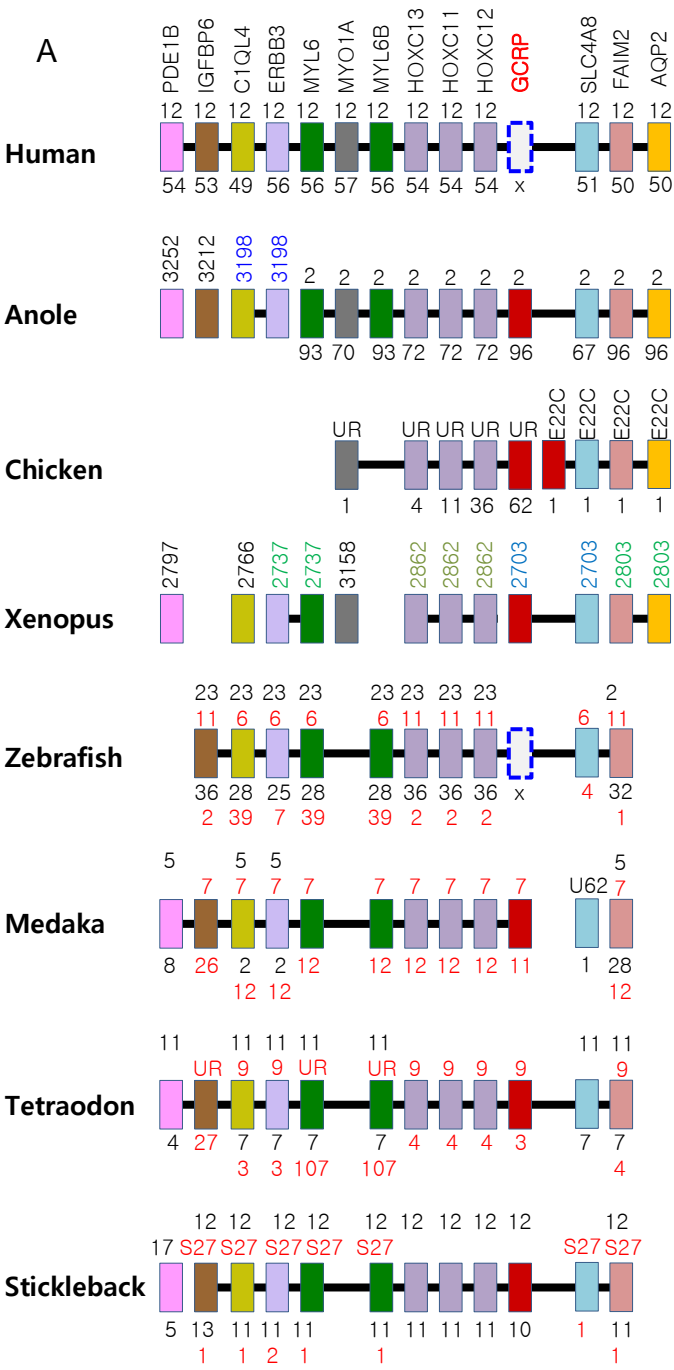

**B**

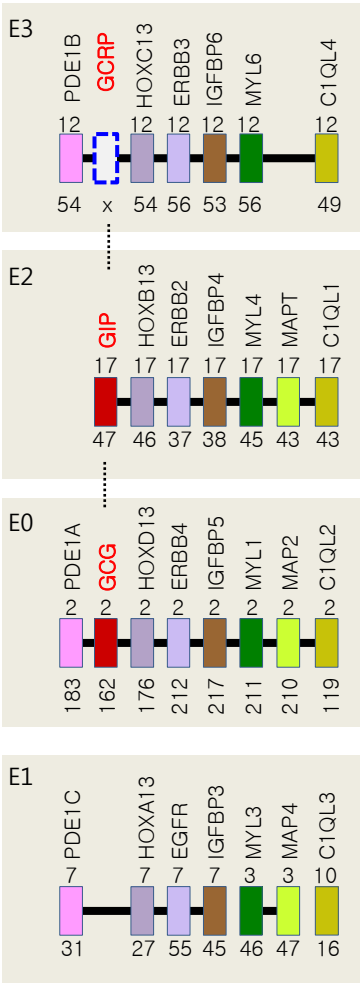

Supplement: Figure S1 — Synteny for the GCRP family gene-containing chromosomes of vertebrates. A, The genomic locations of the GCRP gene and its neighboring genes were compared with those of their orthologous genes in vertebrates, including human, anole, chicken, Xenopus, zebrafish, medaka, tetraodon, and stickleback. Orthologous genes are aligned in the same column. Chromosome numbers are labeled above the indicated gene. Chromosomal locations (megabases) are shown beneath the gene. The genome scaffold numbers for Xenopus started with the same GL17 followed by four variable digits. Only the variable numbers are shown above the gene. For example, if a scaffold number for Xenopus GCRP is GL172703, only 2703 is written above the gene in the figure. Similarly, the last four digits of the genome scaffold numbers for anole are shown. If fish genes were doubled due to teleost-specific genome duplication, the chromosome numbers and gene locations of the doubled genes are indicated in two lines with different colors. The absence of the peptide genes is indicated by white boxes with broken lines, below which ‘x’ is labeled. B, Four putative early paralogons containing the GCRP family peptide gene with their neighboring genes. Human chromosome numbers are above the indicated gene, and the gene locations (megabase) are shown beneath the gene. Paralogs of each gene in different paralogons were aligned on the same column with the same colors. The positions of each gene block in the gnathostome ancestor chromosome (GAC) in the N-models are indicated in the left. (PDF) [file pone.0065420.s001.pdf]

Supplementary Figure S2A

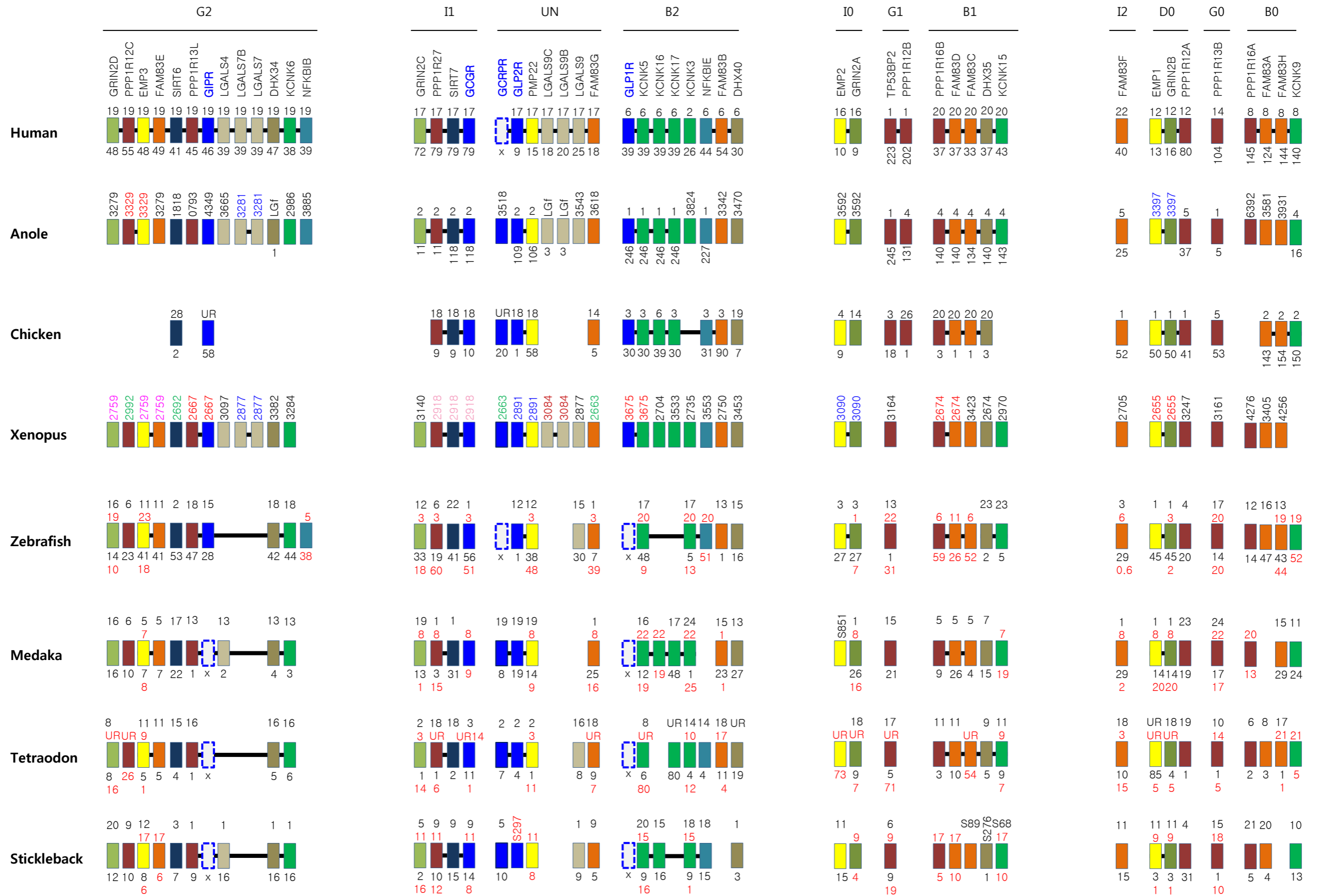

Supplementary Figure S2B

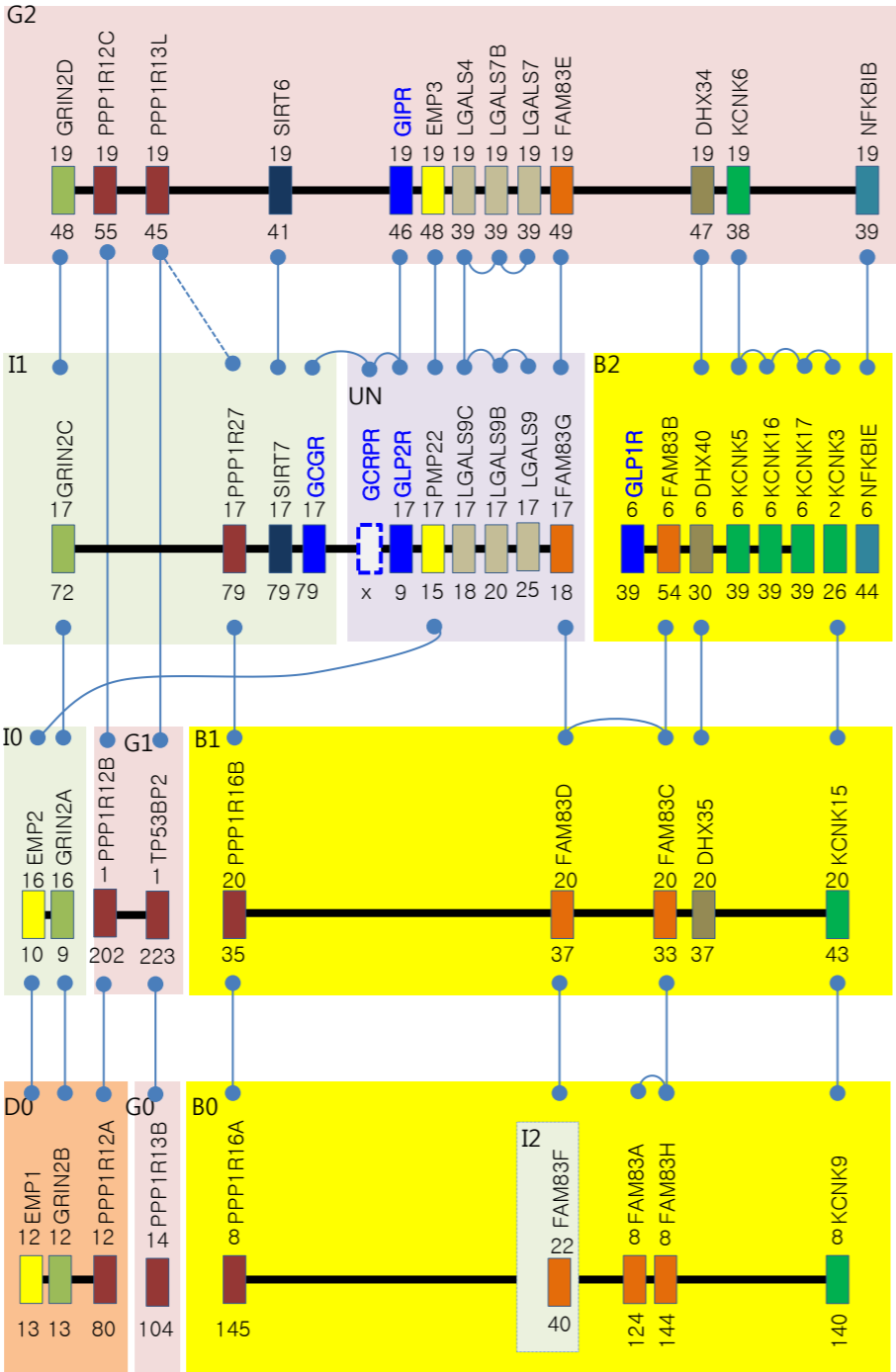

Supplement: Figure S2 — Synteny for the chromosomal locations of GCRPR family genes in vertebrates. A, The genome locations of GCRPR, GCGR, GLP2R, GLP1R, GIPR, and their neighboring genes in vertebrates, including human, anole, chicken, Xenopus, zebrafish, medaka, tetraodon, and stickleback are shown. Orthologous genes are aligned on the same column. Chromosome numbers are shown above the indicated gene, and the gene locations (megabase) are shown beneath the gene. The absence of the receptor genes is indicated by white boxes with broken lines, below which ‘x’ is labeled. The positions of each gene block in the gnathostome ancestor chromosome (GAC) in the N-models are indicated at the top of the column. B, Local duplication of GCGR, GLP2R, GCGPR, and GLP1R after two rounds of whole genome duplication. GIPR and its neighboring genes are aligned on human chromosome 19 of GAC G2. Although GCGR, CCRPR, GLP2R, and GLP1R are on different GAC blocks, they are aligned on one paralogon based on synteny with GIPR-containing paralogon. Two additional paralogons missing GCGR family members were identified by synteny analyses. Human chromosome numbers are shown above the indicated gene, and the gene locations (megabase) are shown beneath the gene. Ohnologs of each gene on different paralogons were aligned on the same column with the same colors and connected by straight lines. Paralogs were connected by a dashed line. Local duplications were indicated by curved lines. (PDF) [file pone.0065420.s002.pdf]
