# Supplementary material for: A Novel Glucagon-Related Peptide (GCRP) and Its Receptor GCRPR Account for Coevolution of Their Family Members in Vertebrates
Source: PLoS One. 2013 Jun 11;8(6):e65420. doi: 10.1371/journal.pone.0065420 (PMC3679108; doi:10.1371/journal.pone.0065420)
Supplement: Information S1 — (PDF) [file pone.0065420.s003.pdf]

Supporting information S1

To determine the evolutionary origin of GCRP and GCRPR and the relationship between their gene families, we analyzed the synteny of GCG-related peptide and receptor genes in the genomes of a variety of vertebrate species. In particular, we analyzed four tetrapod representatives (human, anole, chicken, and *Xenopus*) and four teleost representatives (zebrafish, medaka, tetraodon, and stickleback). Genome synteny analyses were performed by comparing Contig Views of the genomic regions containing the GCG-related peptide and receptor loci. Chromosomal localization of orthologs or paralogs of neighboring genes was obtained from the Ensembl Genome Browser. According to the method of Yegorov and Good (2012), chromosome fragments with reliable synteny were matched with the reconstructed protochromosome models by Nakatani et al. (2007) (herein referred to as the N-model). The origins of the fragments were traced from the gnathostome ancestor chromosome (*GAC*), which contained linkage groups for the hypothetical post-2R ancestor of jawed vertebrates (Nakatani et al., 2007). In the N-model, 40 reconstructed *GACs* (*A0-J1*) are derived from 10-13 vertebrate pre-2R ancestral chromosomes (*VAC*), the *A-J* linkage groups (Nakatani et al., 2007). The N-model provides conserved vertebrate linkage blocks that are displayed along individual human chromosomes, indicating the locations of human ohnologs and orthologs of medaka, chicken, and mouse (Nakatani et al. 2007). Thus, we were able to compare the chromosome fragments with linkage groups shown for each chromosome of the three taxa (human, chicken, and medaka) to resolve the positions of the gene blocks at consecutive stages of vertebrate genome evolution (*e.g.*, pre-2R *VAC* and post-2R *GAC*).

Synteny for GCRP-containing genome fragments and the evolutionary relationship with related genes

The genomic fragments that contain *GCRP* showed conserved synteny across vertebrate species (Supplementary Fig. S1A). *GCRP* was located near phosphodiesterase 1B (*PDE1B*), homeobox C13 (*HOXC13*), v-erb-b2 erythroblastic leukemia viral oncogene homolog 3 (*ERBB3*), insulin-like growth

factor binding protein 6 (*IGFBP6*), myosin light chain 6B (*MYL6B*), and complement component 1 q subcomponent-like 4 (*C1QL6*) in many vertebrates. However, *GCRP* was not located on the human and zebrafish chromosomes that harbored these neighboring genes. There may be two forms of *GCRP* at 62 megabases of Un_random (UR) and at 1 megabase in the E22C19W28 fragment of chickens. These two genes may have emerged by local tandem duplication, because *GCRP* neighbors in chicken E22C19W28, such as aquaporin 2 (*AQP2*), Fas apoptotic inhibitory molecule 2 (*FAIM2*), and solute carrier family 4 sodium bicarbonate cotransporter member 8 (*SLC4A8*) were localized to *GCRP*-containing chromosomes in other vertebrates (Supplementary Fig. S1A). Teleost-specific genome duplication may not have contributed to the development of the second form of *GCRP*. The paralogs (or ohnologs) of *GCRP* neighbors were aligned in parallel in *GIP*- and *GCG*-containing genome fragments (Supplementary Fig. S1B), supporting the concept that *GCG*, *GIP*, and *GCRP* emerged through 2R. According to the N-model, *GCG*, *GIP*, and *GCRP* are localized on *GAC E0*, *E2*, and *E3*, respectively, indicating loss of the fourth form of the *GCG* subfamily in *GAC E1* following 2R but before divergence of teleosts and land vertebrates (Supplementary Fig. S1B).

Synteny for GCRPR-containing genome fragments and the evolutionary relationship with related genes

Synteny analysis showed that *GCRPR* clustered with *GCGR* and *GLP2R* on the same chromosome (Supplementary Fig. S2A). For example, *GCGR* and *GLP1R* were on the same chromosome in human, anole, and chicken and were on either of the teleost-specific duplicated chromosomes of medaka and zebrafish. Because *GCRPR* is found on small scaffold genome fragments in anole, chicken, and *Xenopus*, but absent in human, it is difficult to see co-localization of *GCRPR* with *GCGR* and *GLP2R* on tetrapod chromosomes. However, *GCRPR* and *GLP2R* reside on the same chromosome in some teleost species, such as on Chromosome (Chr) 19 in medaka and Chr 2 in tetraodon. In addition, *Xenopus GCRPR* and family with sequence similarity 83 member G (*FAM83G*) are on scaffold GL272663, whereas *FAM83G* is localized near *GLP2R* on human Chr 17 (Supplementary Fig. S2A).

The presence of *GCRPR*, *GLP2R*, and *GCGR* on the same chromosome in many vertebrates suggests that these genes have arisen through local duplications before or after 2R.

Unexpectedly, *GCGR*, *GIPR*, *GLP2R*, *GRLR*, and *GLP1R* are on different *GAC* blocks according to the N-model (Supplementary Fig. S2B). For instance, *GLP1R* was on *B2*, *GIPR* was on *G2*, *GCGR* was on *I1*, and *GLRL* and *GLP2R* were on *UN* (unassigned). The unexpected locations of the GCGR family members may be due to inaccurate reconstruction of some ancestral linkage groups because of massive chromosomal rearrangement in these regions or a single translocation that caused these genes to move from the original chromosomal fragment following 2R duplication. Thus, the mechanisms by which these family members emerged are likely to be very complex. In this case, small-scale synteny analyses were more useful than large-scale synteny reconstruction of ancestral genomes. Our small-scale synteny analyses revealed that ohnologs (or paralogs) of *GCRPR/GLP2R/GCGR* neighbors, such as glutamate receptor ionotropic N-methyl D-aspartate 2C (*GRIN2C*), protein phosphatase 1 regulatory subunit 27 (*PPP1R27*), sirtuin 7 (*SIRT7*), peripheral myelin protein 22 (*PMP22*), lectin galactoside-binding soluble 9C (*LGALS9C*), and *FAM83E* were found on GIPR-containing genome fragments. These results suggest that *GCRPR/GLP2R/GCGR*- and *GIPR*-containing genome fragments developed by 2R. In addition, ohnologs (or paralogs) of *GIPR* neighboring genes DEAH box polypeptide 34 (*DHX34*), potassium channel subfamily K member 6 (*KCNK6*), and nuclear factor of kappa light polypeptide gene enhancer in B-cells inhibitor beta (*NFKBIB*) were observed in *GLP1R*-containing genome fragments (Supplementary Fig. S2). This observation raise the possibility that one paralogon harboring *GCRPR/GLP2R/GCGR* and *GLP1R* may have split into two chromosomal fragments. Thus, it seems likely that *GCRPR* has emerged through local duplication of an ancestral gene of *GCRPR*, *GLP2R*, *GCGR*, and possibly *GLP1R*. However, further investigation is required.

References

1. Yegorov S, Good S (2012) Using paleogenomics to study the evolution of gene families: origin and

duplication history of the relaxin family hormones and their receptors. PLoS One 7: e32923.

2. Nakatani Y, Takeda H, Kohara Y, Morishita S (2007) Reconstruction of the vertebrate ancestral genome reveals dynamic genome reorganization in early vertebrates. Genome Res 17: 1254-1265.
